# Dynamic Field Monitoring Based on Multitask Learning in Sensor Networks

**DOI:** 10.3390/s19071533

**Published:** 2019-03-29

**Authors:** Di Wang, Xi Zhang

**Affiliations:** Department of Industrial Engineering and Management, Peking University, Beijing 100871, China; di.wang@pku.edu.cn

**Keywords:** multitask learning, field monitoring, missing data, cumulative sum (CUSUM) control chart

## Abstract

Field monitoring serves as an important supervision tool in a variety of engineering domains. An efficient monitoring would trigger an alarm timely once it detects an out-of-control event by learning the state change from the collected sensor data. However, in practice, multiple sensor data may not be gathered appropriately into a database for some unexpected reasons, such as sensor aging, wireless communication failures, and data reading errors, which leads to a large number of missing data as well as inaccurate or delayed detection, and poses a great challenge for field monitoring in sensor networks. This fact motivates us to develop a multitask-learning based field monitoring method in order to achieve an efficient detection when considerable missing data exist during data acquisition. Specifically, we adopt a log likelihood ratio (LR)-based multivariate cumulative sum (MCUSUM) control chart given spatial correlation among neighboring regions within the monitored field. To deal with the missing data problem, we integrate a multitask learning model into the LR-based MCUSUM control chart in the sensor network. Both simulation and real case studies are conducted to validate our proposed approach and the results show that our approach can achieve an accurate and timely detection for an out-of-control state when a large number of missing data exist in the sensor database. Our model provides an effective field monitoring strategy for engineering applications to accurately and timely detect the products with abnormal quality during production and reduce product losses.

## 1. Introduction

Dynamic fields widely exist in engineering systems, which have interactions among regions due to spatial correlation in the space domain and run over time. Field monitoring plays a critical role in determining whether a dynamic process is in a state of statistical process control. In recent years, field monitoring has been widely applied to various engineering domains, including epidemic disease surveillance [1], solar flare detection [2], machine health monitoring [3], and computer network intrusion detection [4].

An out-of-control incident in dynamic field often occurs along with a cluster of neighboring regions because dynamic fields generally present with a spatial correlation. Multiple sensor observations are successively collected from distributed sensor networks (DSNs) developed on the field to monitor such a dynamic process. Given this scenario, the decision maker can simultaneously monitor numerous neighboring regions across the field by analyzing the sensor observations to detect when and where an out-of-control cluster occurs, which is important in engineering applications for monitoring product quality and reducing product losses. For example, an increase in flu outbreak often occurs in a cluster of regions during influenza season. Once an outbreak cluster occurs in certain regions, triggering alarms from the observed data as quickly as possible and then identifying the regions where an outbreak has occurred are critical for the disease control center. Another example can be found in grain storage. An overheat cluster of grain temperature often occurs in a granary due to grain mildew and pests, which indicates that the grains have deteriorated. If the overheat cluster is untimely detected, it will propagate around to become a large overheat cluster, which leads to substantial grain losses.

Dynamic field monitoring is based on sensor observations collected from DSNs in the monitored space domain. One of the major challenges for monitoring the dynamic field is the limited sensor observations available in engineering practice. This issue is caused by sensor observations not being gathered appropriately into a single database for some unexpected reasons such as sensor aging, wireless communication failures, and data reading errors. Consequently, a large number of missing values are generated among the sensor observations. Therefore, developing an efficient monitoring method for the dynamic field when only a limited number of sensor observations are accessible is necessary.

### 1.1. Related Work

#### 1.1.1. Control Charts for Field Monitoring

In recent years, different types of the control chart have been extensively adopted for monitoring dynamic fields and detecting regional outbreaks. The main purpose of control charts is to detect the out-of-control state in an early stage while simultaneously preventing false alarms. Shewhart charts borrow the mean, range, or proportion of observations as statistics and detect an outbreak on the basis of the upper and lower limits that are determined by the statistic dispersion [5]. This pioneering process detection tool is adept for detecting large shifts of a process but fails to distinguish small ones. Exponentially weighted moving average (EWMA) and cumulative sum (CUSUM) charts have been proposed to improve the performance of small shift detection. EWMA charts use the exponentially-weighted moving average of observations [6] while CUSUM charts use cumulative sums of observations [7]. In comparison with EWMA charts, CUSUM charts indicate a higher sensitivity to small shifts because shift information can be accumulated during the entire monitoring time period by a recursive procedure.

CUSUM charts serve as a popular tool in many process monitoring methods, such as detecting a slowly emerging disease cluster that usually does not cause a sudden, large shift in the number of disease counts [8,9]. Page adopted a univariate CUSUM chart for monitoring a time-varying process in which only a single region is monitored at each time point [10]. Fricker et al. compared the performance of a univariate CUSUM chart with two Shewhart charts and one EWMA chart and found that the CUSUM chart significantly outperformed the other charts [11]. The univariate CUSUM charts have been widely applied for monitoring a time variant process in various engineering domains. However, these charts cannot be used for monitoring a dynamic process that varies across space and time because they are constructed on the basis of observations of a single region. Thus, interactions among regions due to spatial correlation cannot be reflected.

In the previous decades, multivariate CUSUM (MCUSUM) charts have been presented by researchers for field monitoring to monitor observations in a cluster of regions [12,13]. Rogerson and Yamada adopted an MCUSUM chart that used local statistics defined as a weighted sum of observations in a local cluster to detect an outbreak cluster [14]. Sonesson extended Rogerson and Yamada’s work and defined a spatial cluster as a group of regions in a circle [15]. However, spatial correlation among regions in the circle was ignored. To fill this gap, some researchers have adopted MCUSUM charts for detecting an outbreak by considering spatial correlation among regions. These MCUSUM charts use observations to formulate statistics including Hotelling’s T^2^ and log likelihood ratio (LR) statistics. Boullosa-Falces et al. obtained a Hotelling’s T^2^ statistics using observations at each time point and then formed a CUSUM chart based on a time sequence of Hotelling’s T^2^ statistics [16]. However, Hotelling’s T^2^ chart uses a global statistic based on observations of the entire space domain instead of local statistics of spatial clusters. To construct local statistics of spatial clusters, Jiang et al. proposed an LR-based MCUSUM chart, which developed an LR statistic for each spatial cluster and scanned all clusters for detecting an underlying outbreak [17]. Lee et al. extended Jiang’s work by adopting an analytical formula to approximate the control limit in the LR-based MCUSUM chart [18].

The previously mentioned MCUSUM charts require sufficient available observations at each time point to detect an outbreak. One of the major challenges for monitoring the dynamic field is that a large number of missing values are generated among the sensor observations. Conventional field monitoring methods may not detect an outbreak cluster in a timely manner once it occurs due to the missing data problem in sensor observations. Other methods for field monitoring, like deep-learning based detection, also cannot handle missing data problem when monitoring a dynamic field [19]. Therefore, it is necessary to develop an efficient monitoring method for the dynamic field when missing data exist in sensor observations. Liu et al. [2] and Xian et al. [20] developed local statistics by introducing a compensation coefficient when sensor observations are unavailable. However, these methods do not effectively work when a large number of sensor observations are unavailable.

#### 1.1.2. Methods for Handling Missing Data Problem in Sensor Observations

To solve the missing data problem, interpolation methods have been adopted to fill in missing values using existing sensor observations, including linear, spline [21], and Lagrange interpolations [22]. However, these strategies may cause large bias and cannot capture interactions in the spatial domain.

Transfer learning provides an opportunity for field monitoring using sensor observations with a missing data problem [23]. In recent years, transfer learning has been studied by numerous researchers and applied to various engineering domains including WiFi localization [24], speech emotion recognition [25], and manufacturing shape deviation [26]. Multitask learning has emerged as one of the popular focuses for transfer learning problems [27]. Compared with Single-task learning [28,29] that learns “knowledge” by using the existing sensor observations in the target process, multitask learning is a machine learning framework that aims to improve the learning of unobserved values in the target process by sharing knowledge or information using existing sensor observations from related processes [30]. In recent years, multitask learning has been studied by numerous researchers and applied to various engineering domains, including traffic flow prediction [31], human action recognition [32], and climate forecast [33]. Yu et al. proposed a multitask learning model for learning Gaussian processes from multiple tasks [34]. However, the spatial information is seldom considered in such a model. Shao et al. adopted a multitask learning model to estimate a 2D-machined surface shape using limited sensor observations from related surface shapes [35]. This model considers the spatial correlation of surface shape and improves the modeling accuracy on the basis of the sensor data of related surface shapes. However, this model mainly focuses on modeling static spatial surfaces but neglects monitoring of the machined surfaces. Shireen et al. proposed a spatiotemporal multitask learning model for performance prediction and failure monitoring of solar panels [36]. This model monitors the solar panels using only the error of the predicted value and the real values of the process. The failure incident can be detected once the error exceeds a pre-specified limit. However, this model does not effectively work for small shift detection.

Existing multitask learning methods can learn unobserved values by capturing spatial correlation and sharing knowledge using limited sensor observations from related tasks. To the best of our knowledge, few studies on multitask learning have been used in field monitoring to address the missing data problem in sensor observations.

### 1.2. The Proposed Model

In conclusion, existing methods have research gaps for dynamic field monitoring. First, existing methods for dynamic field monitoring require sufficient sensor observations to achieve a timely detection when an outbreak occurs. When a large number of missing data exist in sensor observations, detection using these methods is inaccurate and delayed. Second, few methods consider spatial correlation when monitoring a dynamic field with limited sensor observations.

In this study, we propose a multitask learning-based field monitoring approach to detect an out-of-control cluster when considerable missing values exist while collecting sensor observations. An outbreak occurs in a cluster of neighboring regions and the radius of the cluster is unknown. Thus, we adopt an LR-based MCUSUM control chart by considering spatial correlation among regions. This chart defines a spatial cluster as a group of regions in a circle with a varying radius and scans all possible spatial clusters. We integrate a multitask learning model into the LR-based MCUSUM control chart to handle the missing data problem in sensor observations. The multitask learning model learns missing values of the target process by sharing knowledge using existing sensor observations from related processes. This dynamic field monitoring tool is particularly designed when partial data from the target process are missing and those from other related processes are accessible. Furthermore, we introduce our research methodology by considering a 2D dynamic process, which is common in engineering practices. Our proposed approach can also be extended to 3D dynamic processes.

The contribution of the proposed model for dynamic field monitoring includes the following aspects. First, a multi-task learning-based monitoring approach is proposed to handle a missing data problem in sensor observations. Second, we adopt an LR-based MCUSUM control chart for dynamic field monitoring by considering spatial correlation among regions.

The remainder of this paper is organized as follows. Section 2 introduces the methodology of multitask learning-based field monitoring. Section 3 and Section 4 present simulation and real-case studies to evaluate the model performance, respectively. Section 5 provides a conclusion.

## 2. Research Methodology

We propose a multitask-learning based field monitoring method to achieve an efficient detection when considerable missing data exist while collecting sensor observations. We consider a 2D dynamic process with a spatial correlation in a 2D space. We aim to monitor this dynamic field using the sensor data collected from DSNs and trigger an alarm once an outbreak cluster occurs in certain regions. Sensor data can be simultaneously collected from L related 2D processes through DSNs, and missing data can occur at any sensor location and time point t.

**Figure 1:**
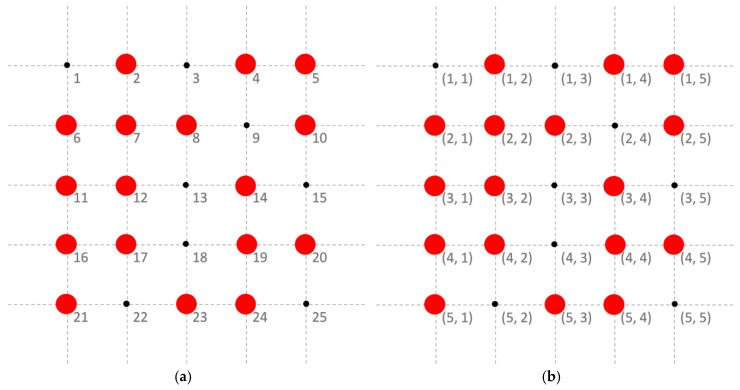
An example of vector and coordinate expressions of sensor locations. (Note: sensor data are observable at the locations with red dots, and missing at the remaining locations.). (**a**) *i*; (**b**) $s_{i}=\left(p,\;q\right)$.

For monitoring target dynamic process $l^{*}$, we suppose that $n=N_{p}\times N_{q}$ sensor locations exist in the space domain. We also define a sensor location by $s_{i}=\left(p,\;q\right)$ with $p=1,\;\ldots ,\;N_{p}$ and $q=1,\;\ldots ,N_{q}$, which represent 2D location coordinates with i=1, …, n. Figure 1 shows an example of the vector and coordinate expressions of sensor locations, in which we set $N_{p}=N_{q}=5$ and, thus, n=5×5=25. Sensor data are ideally collected at each location. We denote the sensor values of process $l^{*}$ at time t as $x_{t}^{l^{*}}={\left(x_{t}^{l^{*}}\left(s_{1}\right),\;\ldots x_{t}^{l^{*}}\left(s_{i}\right),\;\ldots ,\;x_{t}^{l^{*}}\left(s_{n}\right)\right)}^{T}$. In practice, only partial data are observable (e.g., sensor data at the locations with red dots in Figure 1), and the remaining data cannot be successfully collected. We consider the observed sensor values of process $l^{*}$ at time t as ${\tilde{x}}_{t}^{l^{*}}={\left(x_{t}^{l^{*}}\left({\tilde{s}}_{1}\right),\;\ldots x_{t}^{l^{*}}\left({\tilde{s}}_{i}\right),\;\ldots ,\;x_{t}^{l^{*}}\left({\tilde{s}}_{n_{t}^{l^{*}}}\right)\right)}^{T}$, where ${\tilde{s}}_{i}$ denotes the location of the *i*th observed sensor value, and $n_{t}^{l^{*}}$ is the number of observed values of process $l^{*}$ at time t. $n-n_{t}^{l^{*}}$ missing values are observed at time t for process $l^{*}$. The missing data problem poses a considerable challenge for monitoring the dynamic process for detecting an out-of-control cluster. To address this issue, we initially adopt a multitask learning model to estimate the missing values of the monitored process $l^{*}$ by considering the observed sensor values of L related processes to obtain the values at each sensor location of the monitored process. Then, we monitor the process using an LR-based MCUSUM control chart by considering the spatial correlation among different regions in the space domain.

Two assumptions are made prior to the introduction of the proposed model.

**Assumption** **1.** *The* L *related processes demonstrate identical sensor networks and the set of distinct locations of observed values in process* l *, with* l=1, …, L *, can cover all the locations in the sensor networks.*

**Assumption** **2.** *Sensor values are spatially correlated for each process* l *, with* l=1, …, L *.*

On the basis of Assumption 2, we assume that the sensor value vectors $x_{1}^{l^{*}},\;x_{2}^{l^{*}},\;\ldots ,x_{t}^{l^{*}},\;\ldots \;$ of the target process are independently and identically distributed and follow a multivariate normal distribution with a mean vector μ and a covariance matrix Σ. In addition, we assume that the sensor values have been standardized without loss of generality.

For the target process, we assume that $\mu =\mu_{0}$ for all sensor locations $s_{i}$ with i=1, …, n, when no out-of-control clusters occur while $\mu =\mu_{1}$ when an out-of-control cluster occurs. Therefore, we formulate the field monitoring problem for the target dynamic process by testing the null hypothesis as follows.

**H0.**  *No out-of-control spatial clusters exist for all time* t *(i.e.,* $\mu =\mu_{0}$ *, with all the mean of sensor values equal to* $\mu_{0}$ *when* t=1, 2, … *against the composite alternative hypothesis).*

**H1.**  *An emerging out-of-control spatial cluster occurs from time* v *(i.e.,* $\mu =\mu_{0}$ *, with all the mean of sensor values equal to* $\mu_{0}$ *when* t=1, 2, …, v−1 $\mu =\mu_{1}$ *, with the mean of some sensor values equal to* $\mu_{1}$ *, and the others equal to* $\mu_{0}$ *, when* t=v, v+1, … *).*

Here, v is an unknown change point time. $\mu_{0}$ is assumed to be known. We denote $\mu_{1}$ as homogeneous outbreaks, in which a cluster of some sensor values has an identical mean shift magnitude $\mu_{1}-\mu_{0}$. In many engineering applications, the cases that $\mu_{1}$ is component-wise not less than $\mu_{0}$ (i.e., $\mu_{1}\geq \mu_{0}$), which have attracted considerable attention. Thus, we consider $\mu_{1}\geq \mu_{0}$ in our model.

In our model, we develop an effective field monitoring method by integrating a multitask learning model into the LR-based MCUSUM control chart to handle the missing data problem in sensor observations, where the multitask learning model learns missing values of the target process by sharing knowledge using existing sensor observations from related processes and the LR-based MCUSUM control chart is adopted by considering spatial correlation among the regions. We introduce our proposed model in the next subsections in detail.

### 2.1. Multitask Learning for Estimation of Missing Values

**Figure 2:**
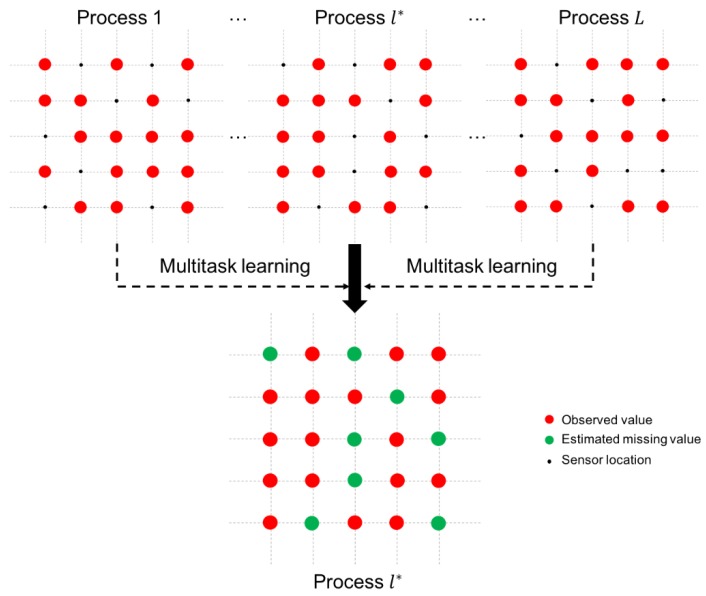
Multitask learning for estimating missing values.

As shown in Figure 2, we consider L-related processes, which include the target process $l^{*}$. We adopt a multitask learning model to estimate the missing values in the target process $l^{*}$ by using the observed sensor values in the L-related processes. At each time t, we denote the latent function of process l as $y_{t}^{l}\left(s\right)$, with l=1, …, L. Given that noises exist in the sensor values, we have $x_{t}^{l}\left(s\right)=\;y_{t}^{l}\left(s\right)+\epsilon_{t}^{l}\left(s\right)$, where $\epsilon_{t}^{l}\left(s\right)$ is assumed to be Gaussian white noise with zero mean and variance $\sigma_{t}^{2}$ (i.e., $\epsilon_{t}^{l}\left(s\right)\sim N\left(0,\;\sigma_{t}^{2}\right)$. We represent the accessible data of process l, with l=1, 2, …, L, as $D_{t}^{l}=\left\{{\tilde{x}}_{t}^{l},S_{t}^{l}\right\}$, where ${\tilde{x}}_{t}^{l}$ denotes the vector of observed values of process $l^{*}$ at time t, and $S_{t}^{l}$ is the set of the locations of observed values at time t of process l. A vector in terms of $y_{t}^{l}\left(s\right)$ is denoted as $y_{t}^{l}={\left(y_{t}\left(s_{1}\right),\;y_{t}\left(s_{2}\right),\;\ldots ,\;y_{t}\left(s_{n}\right)\right)}^{T}$. We assume that $y_{t}^{1},\;y_{t}^{2},\;\ldots ,\;\mathrm{and}\;y_{t}^{L}$ share a common mean and covariance matrix of the Gaussian process because the considered dynamic processes have nearly identical environment and the same operating condition. Furthermore, we assume $y_{t}^{l}$, with l=1, …, L, to be a Gaussian process $y_{t}^{l}\sim GP\left(m_{yt},\;C_{yt}\right)$, which is characterized by a mean vector $m_{yt}$ and a covariance matrix $C_{yt}$. To capture spatial correlation, we assume that $y_{t}^{1}$, $y_{t}^{2}$, …, and $y_{t}^{L}$ share a common structure by modeling the parameters of the Gaussian process on the basis of a spatial kernel κ (i.e., $m_{yt}=\kappa m_{t}$ and $C_{yt}=\kappa C_{t}\kappa^{T}$) where the kernel matrix κ is obtained by a spatial kernel function shown below.

(1) $$\kappa \left(s_{i},s_{j}\right)=\exp\left(-\frac{\|s_{i}-s_{j}\|{}^{2}}{\delta^{2}}\right),$$

where $s_{i}$ and $s_{j}$ are two different sensor locations and δ is the range parameter that corresponds to the distance around the space domain and can be determined as the maximum length of the space domain. Therefore, for $y_{t}^{l}$ with l=1, …, L, a unique $\;\alpha_{t}^{l}$ exists, such that, $y_{t}^{l}=\kappa \alpha_{t}^{l}$, where $\alpha_{t}^{l}$ denotes a vector of weight parameters for process l and $\alpha_{t}^{l}\sim N\left(m_{t},\;C_{t}\right)$, and $m_{t}$ and $C_{t}$ represent the mean vector and covariance matrix of $\alpha_{t}^{l}$, respectively. To obtain the maximum likelihood estimate of $m_{t}$ and $C_{t}$, we use a hyper-prior distribution of $m_{t}$ and $C_{t}$ by a normal-inverse-Wishart distribution, as shown in the formula below.

$$\left\{m_{t},C_{t}\right\}\sim N\left(m_{t}|0,\;\frac{1}{\pi }C_{t}\right)IW\left(C_{t}|\tau ,\;\kappa^{-1}\right),$$

where $m_{t}$ is specified by a multi-normal distribution with a zero mean and a covariance matrix $\left(1/\pi \right)C_{t}$, I and π is a precision, and $C_{t}$ is specified by an inverse-Wishart distribution with precision τ and kernel matrix κ.

We utilize a multitask learning procedure for missing data estimation as follows:

(1) **m***_t_* and **C***_t_* are initiated by the previously mentioned normal-inverse-Wishart distribution:
(2) For *l* = 1, …, *L*, we acquire $\alpha_{t}^{l}$ by $\alpha_{t}^{l}\sim N\left(m_{t},\;C_{t}\right)$;
(3) For *l* = 1, …, *L*, we acquire $y_{t}^{l}=\kappa \alpha_{t}^{l}$.

We now introduce the detailed procedure of the multi-task learning model. We apply an expectation–maximum algorithm to estimate parameters $\alpha_{t}^{l}$, $m_{t}$, and $C_{t}$ at each time t as follows:

*E-step*: For each process l, with l=1, …, L, the expectation and covariance matrix of $\alpha_{t}^{l}$ are estimated using the following current parameter values:

(2) $$\alpha_{t}^{l}={\left(\frac{1}{\sigma_{t}^{2}}\kappa_{l}^{T}\kappa_{l}+C_{t}^{-1}\right)}^{-1}\left(\frac{1}{\sigma_{t}^{2}}\kappa_{l}^{T}{\tilde{x}}_{t}^{l}+C_{t}^{-1}m_{t}\right),$$

(3) $$C_{t}^{l}={\left(\frac{1}{\sigma_{t}^{2}}\kappa_{l}^{T}\kappa_{l}+C_{t}^{-1}\right)}^{-1},$$

where $\kappa_{l}\in {\mathbb{R}}^{n_{t}^{l}\times n}$ is the kernel matrix between $S_{t}$ and $S_{t}^{l}$ obtained by the kernel function in Equation (1), in which $S_{t}$ denotes the set of distinct locations of observed values in all of the L processes ${\left\{S_{t}^{l}\right\}}_{l=1}^{L}$.

*M-step*: $m_{t}$, $C_{t}$, and $\sigma_{t}^{2}$ are optimized on the basis of the last E-step. Thus, the updated values of $m_{t}$, $C_{t}$, and $\sigma_{t}^{2}$ are obtained by using the equations below.

(4) $$m_{t}=\frac{1}{\pi +L}\sum_{l=1}^{L}\alpha_{t}^{l},$$

(5) $$C_{t}=\frac{1}{\tau +L}\left(\pi m_{t}m_{t}^{T}+\tau \kappa^{-1}+\sum_{l=1}^{L}C_{t}^{l}+\sum_{l=1}^{L}\left(\alpha_{t}^{l}-m_{t}\right){\left(\alpha_{t}^{l}-m_{t}\right)}^{T}\right),$$

(6) $$\sigma_{t}^{2}=\frac{1}{\sum_{l=1}^{L}n_{t}^{l}}\sum_{l=1}^{L}\|{\tilde{x}}_{t}^{l}-\kappa_{l}\alpha_{t}^{l}\|{}^{2}+\mathrm{tr}\left(\kappa_{l}C_{t}^{l}\kappa_{l}^{T}\right),$$

where tr(·) denotes the trace of a matrix. We obtain the estimated parameters ${\hat{\alpha }}_{t}^{l^{*}}$, ${\hat{m}}_{t}$, and ${\hat{C}}_{t}$ for process $l^{*}$ by implementing the expectation–maximum algorithm. Then, $y_{t}^{l^{*}}$ is estimated by the equation below.

(7) $${\hat{y}}_{t}^{l^{*}}=\kappa {\hat{\alpha }}_{t}^{l^{*}},$$

where ${\hat{y}}_{t}^{l^{*}}$ denotes the estimate of $y_{t}^{l^{*}}$. Therefore, the estimated missing values of process $l^{*}$ can be obtained from ${\hat{y}}_{t}^{l^{*}}$.

### 2.2. LR-Based MCUSUM Control Chart for Detection

After estimating the missing values, we obtain a complete set of sensor values of process $l^{*}$ as ${\hat{x}}_{t}^{l^{*}}$, which is composed of the observed and estimated missing values. We now adopt an LR-based MCUSUM control chart to monitor process $l^{*}$ and trigger the alarm as soon as possible when an outbreak cluster occurs.

**Figure 3:**
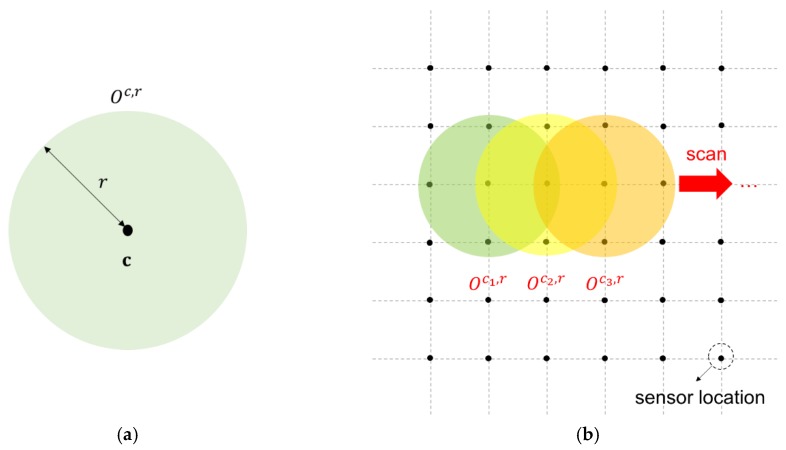
Illustration of the monitoring procedure for local spatial clusters using the spatial scanning method. (**a**) A local spatial cluster. (**b**) The spatial scanning procedure.

We define a local spatial cluster for monitoring the outbreak cluster on the basis of the assumption in Reference [17] where the outbreak cluster is a circle. We denote c and r as the center location and radius of the local cluster, respectively, and define a set of all possible local spatial clusters in the space domain as $O^{c,r}=\left\{s_{i}|\|s_{i}-c\|\leq r\right\}$, where ‖·‖ denotes the Euclidean distance between $s_{i}$ and c. Figure 3a shows an illustration of a local spatial cluster. We consider the local spatial clusters with varying radius r bounded by a given upper limit $r_{u}$ because the size of outbreak clusters is unknown. The distance between sensor locations $s_{i}$ and c in $O^{c,r}$ changes at certain values because alternative locations of $s_{i}$ and c are fixed. Thus, we consider a finite number of possible values for r in set $R=\left\{r_{1},\;r_{2},\;\ldots ,\;r_{u}\right\}$, where u is the total number of possible values for r. We divide the entire monitoring space domain into overlapping local spatial clusters within a subset of sensor locations. Subsequently, we create an LR-based statistic for each local cluster $\;O^{c,r}$ and use a spatial scanning method to scan all local clusters and detect whether the mean level of the regions in some cluster is shifted (Figure 3b). Spatial scanning is achieved by zeroing out the part of the out-of-control mean vector $\mu_{1}$ that falls out of the regions of a cluster $O^{c,r}$ and forms mean vector $\mu_{c,r}$ for cluster $O^{c,r}$. A different mean vector $\mu_{c,r}$ is considered for various local cluster choices in $O^{c,r}$, which is defined by ${\left[\mu_{c,r}\right]}_{j}={\left[\mu_{1}\right]}_{j}$ for all $j\in O^{c,r}$ and 0, otherwise. Then, we calculate the LR-based statistics of all possible clusters in parallel to detect the occurrence of an outbreak.

When an outbreak cluster occurs, a shift at the sensor locations will be implemented within the cluster. We introduce our method in two situations when the shift magnitude $\mu_{1}-\mu_{0}$ is known (i.e., $\mu_{1}$ is known) and when the shift magnitude $\mu_{1}-\mu_{0}$ is unknown.

#### 2.2.1. Known Shift

An MCUSUM chart based on LR statistics is used to detect a shift from $\mu_{0}=0$ to $\mu_{1}$ by considering the spatial correlation among sensor locations. For a local cluster with center c and radius r at time t, an LR statistic is given by the equation below.

(8) $$\ell_{t}^{c,r}=\mu_{c,r}^{T}\Sigma^{-1}\left({\hat{x}}_{t}^{l^{*}}-\frac{\mu_{c,r}}{2}\right),$$

where $\ell_{t}^{c,r}$ is the LR statistic for the local cluster with center c and radius r at time t. Then, the LR-based MCUSUM chart is given by the equation below.

(9) $$S_{t}^{c,r}=\max\left\{0,S_{t-1}^{c,r}+\ell_{t}^{c,r}\right\},\;t=1,\;2,\;\ldots ,$$

where $S_{t}^{c,r}$ is the statistic of the LR-based MCUSUM chart for the local cluster with center c and radius r at time t. At the initial time (i.e., t=0), $S_{0}^{c,r}=0$. We scan across all possible local spatial clusters and calculate the corresponding LR-based MCUSUM statistics in parallel. We also form a global detection statistic $U_{t}$ by taking the maximum of all the statistics, as shown in the formula below.

(10) $$U_{t}=\max_{c}\max_{r}\;S_{t}^{c,r}.$$

An out-of-control cluster is detected whenever $U_{t}$ exceeds a prespecified control limit h (i.e., $U_{t}>h$). Furthermore, h is specified based on the requirement for the in-control average run length $ARL_{0}$ through Monte Carlo simulations.

In this paper, we introduce the in-control and out-of-control average run lengths for determining h. We denote an average in-control run length as $ARL_{0}$, which represents the expected number of states until a false alarm occurs when an in-control process is actually monitored. We denote an out-of-control average run length as $ARL_{1}$, which represents the expected number of states until an alarm occurs when a monitored process is out-of-control. The $ARL_{1}$ value is a commonly used performance measure for timely detection of an out-of-control process. Small $ARL_{1}$ values while possessing a pre-specified large $ARL_{0}$ is desirable for the LR-based MCUSUM chart procedure. We determine the control limit h given a specific value of $ARL_{0}$ through Monte Carlo simulations of in-control processes. Specifically, the determined h can make $ARL_{0}$ equal to the specific value.

#### 2.2.2. Unknown Shift

In engineering applications, $\mu_{1}$ is occasionally unknown and must be specified on the basis of expert knowledge or estimation from data. Although $\mu_{1}$ is unknown, the shift value of $\mu_{1}$ from $\mu_{0}$ is one of a set of alternative given directions in most engineering cases (i.e., $\mu_{1}\in {\left\{\mu_{1}^{k}\right\}}_{1\leq k\leq K}$. We calculate an LR statistic $\ell_{t}^{k,\;c,r}$ for the local cluster with center c and radius r at time t in each direction $\mu_{1}^{k}$, with k=1, …, K, as shown below [37].

(11) $$\ell_{t}^{k,\;c,r}={\left(\mu_{c,r}^{k}\right)}^{T}\Sigma^{-1}\left({\hat{x}}_{t}^{l^{*}}-\frac{\mu_{c,r}^{k}}{2}\right),$$

where $\mu_{c,r}^{k}$ is defined by ${\left[\mu_{c,r}^{k}\right]}_{j}={\left[\mu_{1}^{k}\right]}_{j}$ for all $j\in O^{c,r}$ and 0 otherwise. Then, we set $S_{t}^{k,c,r}$ as the statistic of the LR-based MCUSUM chart for the local cluster with center c and radius r at time t in the direction $\mu_{1}^{k}$, as shown below.

(12) $$S_{t}^{k,c,r}=\max\left\{0,S_{t-1}^{k,c,r}+\ell_{t}^{k,c,r}\right\},\;t=1,\;2,\;\ldots ,$$

and $S_{0}^{k,c,r}=0$ at the initial time. We calculate a global detection statistic by taking the maximum of LR-based statistics at all directions of $\mu_{1}$, as shown below.

(13) $$V_{t}=\max_{k}\max_{c}\max_{r}\;S_{t}^{k,\;c,r}.$$

When $V_{t}>h$, we trigger an alarm for the occurrence of an out-of-control cluster. Evidently, $U_{t}=V_{t}$ when the direction of $\mu_{1}$ is determined.

## 3. Simulation Study

**Figure 4:**
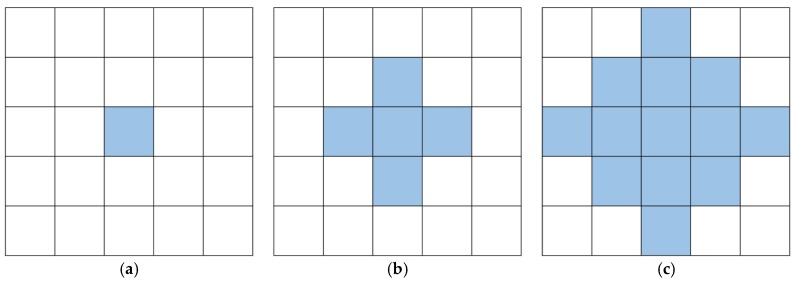
Various patterns of the outbreak cluster. (**a**) Type 1: small cluster; (**b**) Type 2: medium cluster; (**c**) Type 3: large cluster.

We simulate a spatiotemporal process with n=5×5 locations in the space domain. By assuming the spatiotemporal process is spatially correlated, we simulate 300 time points and generate data at each time point in accordance with the multivariate normal distribution $N\left(\mu_{0},\;\Sigma \right)$, where we set $\mu_{0}=0$ as a 25×1 mean vector and Σ as a 25×25 covariance matrix. The covariance matrix is generated from a covariance function $c\left(s_{i},s_{j}\right)=\exp\left(-\|s_{i}-s_{j}\|{}^{2}/d\right)$, where $s_{i}$ and $s_{j}$ are the locations of two different locations i and j, respectively, and the scale parameter d is set to 250. We consider that an out-of-control outbreak cluster occurs at time 51 and remains to the end. Moreover, we assume that the outbreak cluster exhibits a homogeneous shift magnitude with $\mu_{1}$ and consider various patterns of the outbreak cluster (Figure 4a–c). In addition, we set three shift magnitude states, that is, small, medium, and large shift states, corresponding to *μ*_1_ = 0.5, 1, and 2, respectively.

We consider three related spatiotemporal processes (i.e., l=1, 2, 3) and regard $l^{*}=1$ as the target process to be monitored. On basis of the preceding generated data, we obtain the values of each spatiotemporal process with l=1, 2, 3 by adding a noise term that follows a normal distribution $N\left(0,\;0.1^{2}\right)$ on the generated data. We consider that the three related processes demonstrate different missing data patterns by randomly selecting data from the values of each spatiotemporal process and assuming that these data are missing. Furthermore, we set the ratios of the missing data to the entire data value of each process as 20%, 30%, and 50% at each time t. Therefore, we obtain the observed data set of process l at each time t as ${\tilde{x}}_{t}^{l}={\left(x_{t}^{l}\left({\tilde{s}}_{1}\right),\;\ldots x_{t}^{l}\left({\tilde{s}}_{i}\right),\;\ldots ,\;x_{t}^{l}\left({\tilde{s}}_{n_{t}^{l}}\right)\right)}^{T}$ with l=1, 2, 3.

Before evaluating our model performance, we initially specify the control limit h by setting $ARL_{0}$ to 1000. The control limit h for $ARL_{0}=1000$ is determined by using the in-control data generated from the multivariate normal distribution $N\left(\mu_{0},\;\Sigma \right)$ on the basis of 1000 replications. Then, we calculate $ARL_{1}$ values and implement 100 replications for different shift magnitudes, outbreak cluster types, and ratios of missing values. In addition, the results are compared with a benchmark model. Similar to our proposed model, the benchmark model uses an LR-based MCUSUM control chart to detect an out-of-control cluster by considering spatial correlation. The only difference between the benchmark model and our proposed model is that multitask learning is disregarded in the former for handling the missing data problem. The detailed information about the benchmark model can be found in the appendix.

**Figure 5:**
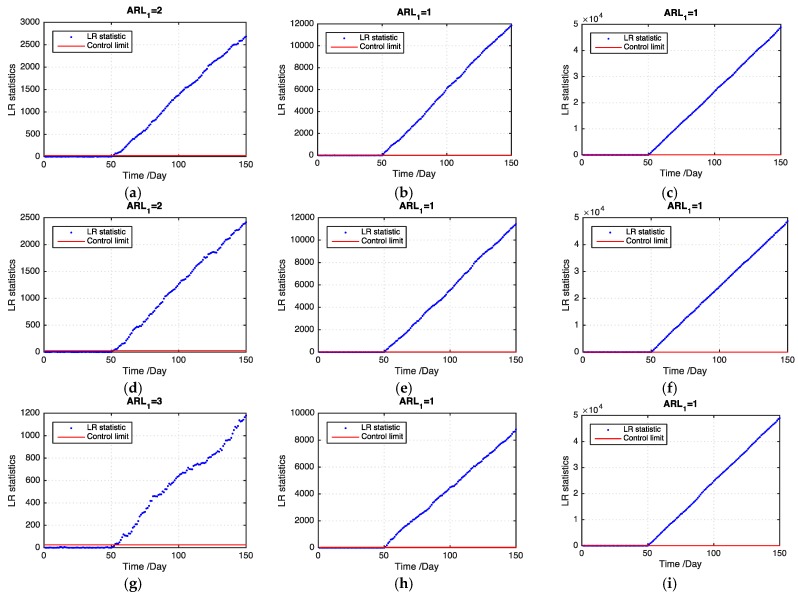
LR statistics using the proposed model for detecting the medium outbreak cluster. (Note: the ranges of the *Y*-axis in each graph are substantially different.). (**a**) shift magnitude = 0.5, 20% missing values; (**b**) shift magnitude = 1, 20% missing values; (**c**) shift magnitude = 2, 20% missing values; (**d**) shift magnitude = 0.5, 30% missing values; (**e**) shift magnitude = 1, 30% missing values; (**f**) shift magnitude = 2, 30% missing values; (**g**) shift magnitude = 0.5, 50% missing values; (**h**) shift magnitude = 1, 50% missing values; (**i**) shift magnitude = 2, 50% missing values.

**Figure 6:**
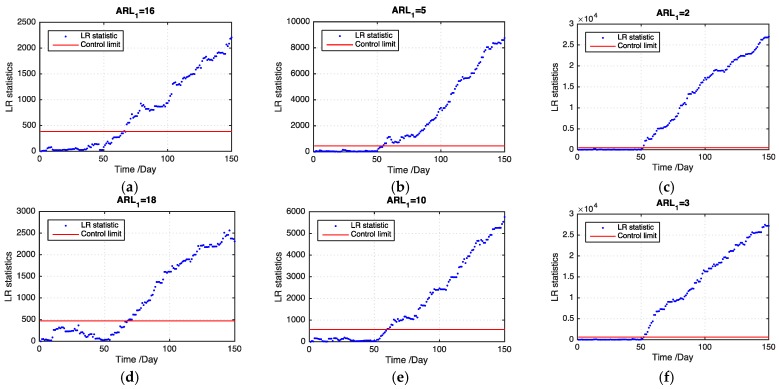

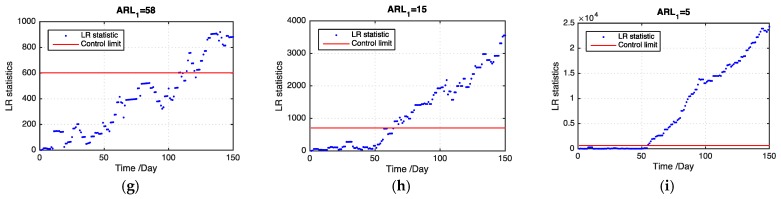
LR statistics using the benchmark model for detecting the medium outbreak cluster. (**a**) shift magnitude = 0.5, 20% missing values; (**b**) shift magnitude = 1, 20% missing values; (**c**) shift magnitude = 2, 20% missing values; (**d**) shift magnitude = 0.5, 30% missing values; (**e**) shift magnitude = 1, 30% missing values; (**f**) shift magnitude = 2, 30% missing values; (**g**) shift magnitude = 0.5, 50% missing values; (**h**) shift magnitude = 1, 50% missing values; (**i**) shift magnitude = 2, 50% missing values.

Table 1 presents the $ARL_{1}$ values of the two models on the basis of 100 replications. Evidently, our proposed model outperforms the benchmark model by using a multitask learning approach to handle the missing data problem. Particularly, when the ratio of missing values becomes large, our proposed model detects an outbreak cluster considerably faster than the benchmark model. For different types of cluster, the small cluster is more difficult to detect than the large one using both models. However, our proposed model performs better than the benchmark model in detecting the small cluster. For different shift magnitudes, our proposed model shows good performance for detecting small, medium, and large shifts. By contrast, the benchmark model does not perform well for the small and medium shifts and even loses the detection power when the shift magnitude is small. Figure 5 and Figure 6 show examples of the LR statistics at the first 150 time points for detecting the medium outbreak cluster with different shift magnitudes and ratios of missing values using our proposed model and the benchmark model, respectively. From the figures, our proposed model can detect an outbreak cluster more quickly than the benchmark model.

## 4. Real Case Study

In this section, we conduct a case study on monitoring the temperature of the stored grains in a granary to test the performance of our proposed model. Grain storage is a critical issue in the national economy and livelihood of people. Grain quality decreases if grains are inefficiently stored. A total of 8% of grains worldwide are annually lost due to considerable unexpected reasons, according to reports from the Food and Agriculture Organization of the United Nations. Grain quality monitoring is necessary during storage to reduce grain losses. Grain temperature monitoring plays an essential role in grain storage because the temperature is among the key factors that may directly influence the quality of stored grains. When grain quality decreases due to some unexpected reasons, including mildew, pests, and high environmental temperature outside the granary, the grain temperature will simultaneously increase. This is because substantial heat will be released to increase the grain temperature to a high level when mildew and pests destroy the grains. The overheat of the grains will propagate around and formulate an overheated cluster. The overheated cluster should be detected as soon as possible to prevent grain losses.

Grain temperature can be divided into two parts [38]: global temperature trends caused by external factors (e.g., environmental temperature) and local temperature variations caused by internal factors (e.g., mildew and pests). In comparison with global temperature trends, local temperature variations have been given considerable attention by practitioners because such changes usually trigger systematic changes or even system failure. An increase in local temperature due to the overheat by the grain self-breath or mildewing may induce an extensive temperature increase spreading across the granary, which leads to unexpected grain deterioration before releasing the grain processing plants. Local temperature variations play a considerably more essential role than global temperature trends in grain storage systems to provide useful information for the surveillance, maintenance, and improvement of a system. Therefore, we remove the global temperature trends from the sensor observations of grain temperature and obtain the observed values of local temperature variations. The detailed information about the modeling of global temperature trends can be found in Reference [38].

**Figure 7:**
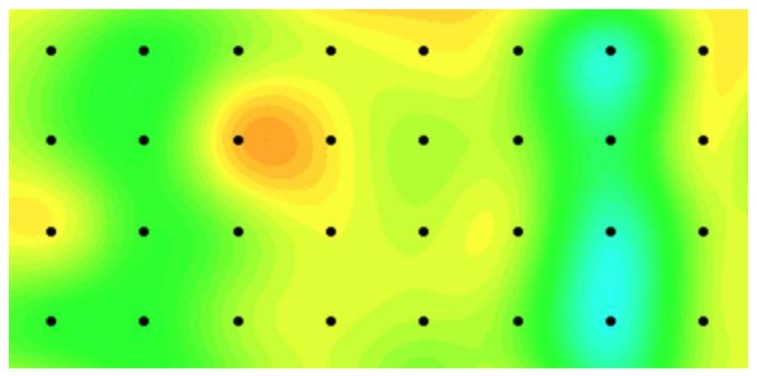
Illustration of a grain temperature plan during grain storage.

We use our proposed model and the benchmark model in the real-case study to monitor local temperature variations of a target granary in a national grain depot located in Central China. We also select two adjacent granaries in the same grain depot, which have an identical structure and store the same grain type. The temperature sensor data of the three granaries are synchronously collected at least every seven days from the sensor networks, and a total of 47 time points exist. Two layers of temperature sensors are set in the granaries and 8 × 4 = 32 evenly spaced sensors are distributed in each layer and located at 5 m intervals between two adjacent sensors. Grain temperature in a granary can be monitored on the basis of the sensor data on each layer. We use sensor data on one of the layers to validate our model performance. Figure 7 presents an illustration of the grain temperature plan during grain storage where the thermal map represents the grain temperature and the black dots indicate the sensor locations. An overheat cluster occurs from the 15th time point because of high environmental temperature. For each granary, we randomly select a number of sensor data and assume that the data are missing. We consider three levels of missing data and set the ratios of the missing data to the entire data as 20%, 30%, and 50% at each time point to evaluate the monitoring efficiency of the proposed model for handling the missing data problem. Furthermore, we repeat the procedure by randomly selecting missing data 100-fold and evaluate the proposed method on the basis of the 100 replications.

**Figure 8:**
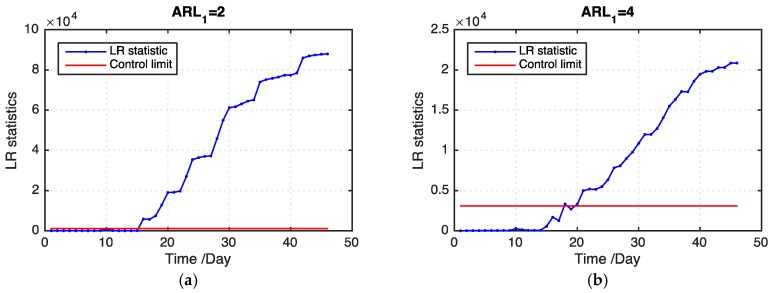

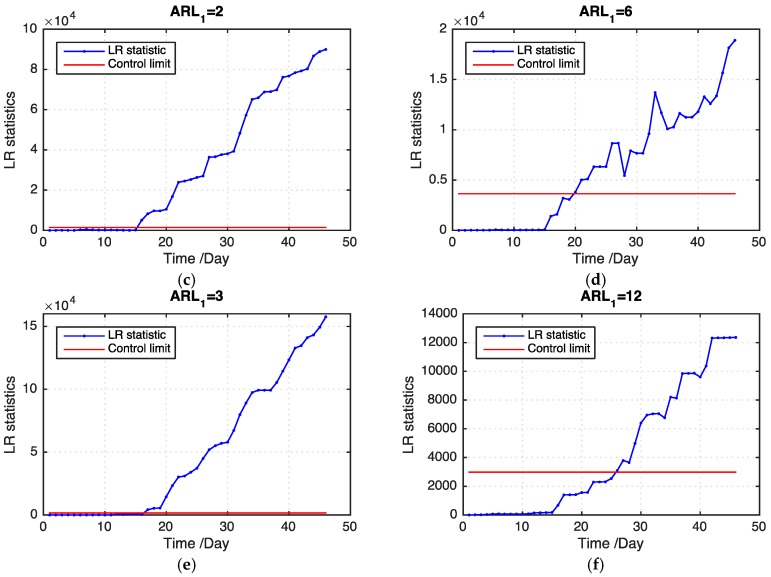
LR statistics using the proposed model and benchmark model for the real case. (**a**) The proposed model includes 20% missing values; (**b**) The benchmark model includes 30% missing values; (**c**) The proposed model includes 30% missing values; (**d**) The benchmark model includes 30% missing values; (**e**) The proposed model includes 50% missing values; (**f**) The benchmark model includes 50% missing values.

$\mu_{1}$ is unknown in the real-case study. Nevertheless, a set of candidate values of $\mu_{1}$ can be obtained by the engineering knowledge of grain storage. We use Equations (11)–(13) to detect an outbreak cluster. Table 2 presents the *ARL*_1_ values of our proposed model and the benchmark model on the basis of 100 replications. Our proposed model outperforms the benchmark model in all levels of missing data. In addition, our proposed model detects an outbreak cluster considerably faster than the benchmark model when the ratio of missing values becomes large. Figure 8 shows examples of the LR statistics for different levels of missing values using our proposed model and the benchmark model. From the figure, our proposed model can detect an outbreak more quickly than the benchmark model. The monitoring results can provide useful information for grain quality assurance in grain storage, which is helpful in reducing grain losses.

## 5. Conclusions

Dynamic field monitoring, which detects an out-of-control event by learning the state change from the collected sensor data, serves as an essential tool in a variety of engineering domains. However, in practice, a large number of missing data exist in the sensor database, which leads to inaccurate or delayed detection when monitoring a dynamic field. The inaccurate or delayed detection causes great losses of products in engineering applications. Therefore, it is essential to develop an effective approach to handle the missing data problem for dynamic field monitoring.

In this study, we propose a multi-task learning-based field monitoring approach to detect an outbreak cluster using sensor data with missing values. We adopt an LR-based MCUSUM control chart by considering spatial correlation among regions to detect an outbreak that usually occurs in a cluster of neighboring regions. Particularly, we integrate a multitask learning model into the LR-based MCUSUM control chart to handle the missing data problem in the sensor data. The multitask learning model learns the missing values of the target process by sharing knowledge or information using observed sensor data from related processes.

The results in simulation and real-case studies show that our model achieves an accurate and timely detection for an out-of-control state when a large number of missing data exist in the sensor database. Our model provides an effective field monitoring strategy for engineering applications to accurately and timely detect the products with abnormal quality during production and reduce product losses.

In our future work, we will establish an effective online monitoring strategy for simultaneously monitoring multiple processes by developing an adaptive sampling approach to determine what sensor values should be observed when only a limited number of sensor resources are available in the space domain.

## Figures and Tables

**Table 1:** *ARL*_1_ values of the proposed model and benchmark model in the simulation case.

			The Proposed Model			Benchmark Model		
The Ratio of Missing Values		Shift Magnitude	0.5	1	2	0.5	1	2
	Outbreak Cluster Type							
20%	Small		5.22	1.54	1.00	40.96	12.61	4.60
	Medium		1.60	1.00	1.00	17.44	6.22	2.14
	Large		1.52	1.00	1.00	15.61	5.66	2.01
30%	Small		6.16	1.68	1.00	51.92	17.82	5.78
	Medium		1.76	1.00	1.00	21.00	8.11	3.00
	Large		1.58	1.00	1.00	20.94	7.18	2.98
50%	Small		12.40	2.92	1.06	101.64	32.94	9.32
	Medium		2.80	1.00	1.00	62.82	18.82	6.08
	Large		2.52	1.00	1.00	44.06	14.79	5.02

**Table 2:** *ARL*_1_ values of the proposed model and benchmark model in the real case.

Model	The Ratio of Missing Values		
	20%	30%	50%
The proposed model	2.45	2.86	3.66
The benchmark model	4.55	5.83	8.42

## Appendix A. Benchmark Model

In order to validate our model performance, we compare our model with a benchmark model [2,17]. The benchmark model uses an LR-based MCUSUM control chart to detect an out-of-control cluster by considering spatial correlation, but multitask learning is disregarded in the benchmark model for handling the missing data problem. The benchmark model interpolates the missing values by zero and obtains a processed data set $z_{t}^{l^{*}}$. The LR-based MCUSUM control chart with known shift is a special case of the one with unknown shift. We just introduce the benchmark model with unknown shift. We consider each sensor location as a center.

When the sensor value at a center c is known, we calculate an LR statistic ${ℊ}_{t}^{k,c,r}$ for the local spatial cluster with radius r at time t in each direction $\mu_{1}^{k}$ with k=1, …, K.

(A1) $${ℊ}_{t}^{k,c,r}={\left(\mu_{c,r}^{k}\right)}^{T}\Sigma^{-1}\left(z_{t}^{l^{*}}-\frac{\mu_{c,r}^{k}}{2}\right),$$

Then, we set the statistic of the LR-based MCUSUM chart $G_{t}^{k,c,r}$ with center c and radius r at time t in the direction $\mu_{1}^{k}$ as shown below.

(A2) $$G_{t}^{k,c,r}=\max\left\{0,G_{t-1}^{k,c,r}+{ℊ}_{t}^{k,c,r}\right\},\;t=1,\;2,\;\ldots ,$$

and $G_{0}^{k,c,r}=0$ at the initial time.

When the sensor value at center c is missing, we construct the statistic $G_{t}^{k,c,r}$ by introducing a compensation coefficient Δ≥0 following the procedure in Reference [2].

$$G_{t}^{k,c,r}=G_{t-1}^{k,c,r}+\Delta ,\;t=1,\;2,\;\ldots ,$$

where Δ is a constant tuning parameter. We set Δ =1 in our simulation and real case studies. Lastly, we calculate a global detection statistic $W_{t}$ by taking the maximum of LR-based statistics at all directions of $\mu_{1}$.

(A3) $$W_{t}=\max_{k}\max_{c}\max_{r}\;G_{t}^{k,\;c,r}.$$

When $W_{t}>h$, we trigger an alarm for the occurrence of an outbreak cluster.
